# *UCHL1*/PGP 9.5 Dynamic in Neuro-Immune-Cutaneous Milieu: Focusing on Axonal Nerve Terminals and Epidermal Keratinocytes in Psoriatic Itch

**DOI:** 10.1155/2018/7489316

**Published:** 2018-07-25

**Authors:** Piotr Kupczyk, Adam Reich, Mariusz Gajda, Marcin Hołysz, Edyta Wysokińska, Maria Paprocka, Dmitry Nevozhay, Grzegorz Chodaczek, Paweł P. Jagodziński, Piotr Ziółkowski, Jacek C. Szepietowski

**Affiliations:** ^1^Department of Pathomorphology, Faculty of Medicine, Silesian Piast Wroclaw Medical University, Wroclaw, Poland; ^2^Laboratory of Immunogenetics and Tissues Immunology, Ludwik Hirszfeld Institute of Immunology and Experimental Therapy, Polish Academy of Sciences, Wroclaw, Poland; ^3^Department of Dermatology, Institute of Experimental and Clinical Medicine, Faculty of Medicine, University of Rzeszow, Rzeszow, Poland; ^4^Department of Histology, Medical Collage, Jagiellonian University, Krakow, Poland; ^5^Department of Biochemistry and Molecular Biology, Karol Marcinkowski Medical University of Poznan, Poznan, Poland; ^6^Laboratory of Tumor Molecular Immunobiology, Ludwik Hirszfeld Institute of Immunology and Experimental Therapy, Polish Academy of Sciences, Wroclaw, Poland; ^7^Laboratory of Glycobiology and Cell Recognitions, Ludwik Hirszfeld Institute of Immunology and Experimental Therapy, Polish Academy of Sciences, Wroclaw, Poland; ^8^School of Biomedicine, Far Eastern Federal University, Vladivostok, Russia; ^9^Department of Imaging Physics, MD Anderson Cancer Center, The University of Texas, Houston, Texas, USA; ^10^Confocal Microscopy Laboratory, Wroclaw Research Centre EIT+, Wroclaw, Poland; ^11^Department of Dermatology, Venereology and Allergology, Silesian Piast Wroclaw Medical University, Wroclaw, Poland

## Abstract

Psoriasis is an immunogenetic skin disease manifesting as plaque lesions on the skin. Patients with psoriasis frequently suffer from itch, an unpleasant sensation causing a desire to scratch. Psoriatic itch is mainly transmitted by unmyelinated C-fibers; however, the exact molecular mechanism of psoriatic itch is still unexplained. Protein gene product 9.5 (PGP 9.5) is a panneurological marker commonly used for analysis of peripheral peptidergic and nonpeptidergic nerves and identification of cutaneous neuro-immune-endocrine cells. However, some studies suggested that nonneuronal cells, like keratinocytes, may also express PGP 9.5. This phenomenon might be linked with impaired axonal transport, keratinocyte injury, or dysfunctions of neuro-immune-cutaneous connections. The aim of this study was to analyze the expression of PGP 9.5 in psoriatic skin. We observed significantly altered density of PGP 9.5-positive axonal nerve terminals in pruritic lesional (p=0.04) and nonlesional psoriatic skin (p>0.001) compared with controls. In contrast, no significant differences were observed between psoriatic skin without itch and controls. Furthermore, PGP 9.5 expression by suprabasal keratinocytes (SBKs) was significantly increased in itchy skin lesions (p=0.007) compared to skin without itch, and a positive correlation was observed between PGP 9.5 expression and itch intensity (r=0.64; p=0.02). Our findings indicate changes in peripheral innervations and psoriatic keratinocytes, which may influence neuro-immune-cutaneous homeostasis and modulate itch transmission.

## 1. Introduction

Psoriasis is an immunogenetic skin disease, in which neuroinflammation is believed to be an important pathogenic element [1]. Neuroinflammation is considered as one of the possible mechanisms which might enhance itch perception, an unpleasant sensation causing a desire to scratch [2, 3]. The neurobiological background of itch in psoriasis is still poorly understood; however abnormalities in peripheral nervous system (PNS) are considered as one of the possible mechanisms [4]. Itch is mainly mediated by unmyelinated C-fibers, which originate from dorsal root ganglion (DRG) neurons [5]. All (peptidergic and nonpeptidergic) PNS structures could be visualized by a neurobiological marker: protein gene product 9.5 (PGP 9.5), one of the most abundant proteins of central nervous system (CNS). Its quantity constitutes up to 3–5% of the total brain protein amount [6, 7]. PGP 9.5 is a ubiquitin carboxyl-terminal hydrolase L1 (UCHL1) with dual hydrolase and ligase activity and molecular weight of 27 kDa, encoded by* UCHL-1* gene [6, 8]. PGP 9.5 is also a deubiquitinating enzyme (DUB) participating in posttranslational protein modification by adding to and removing ubiquitin from polyubiquitin chains [6]. Abnormalities in recycling or prevention of ubiquitinated proteins from the proteosomal degradation pathways are engaged in several neuro-immune-related diseases [6, 8]. For example, the loss of PGP 9.5 protein is linked with decreased mono-Ub pool and results in abnormal function of postsynaptic compartments in PNS terminals [9, 10]. In turn, mutations in* UCHL1* gene are associated with Parkinson disease, engaged in motor neuron circuitry and pain-related dysfunctions [6, 11]. In contrast, overexpression of* UCHL1*/PGP9.5 was documented as protective factor, which delays progression of Alzheimer disease [12]. Moreover,* UCHL1*/PGP 9.5 abnormalities were also linked with cancers, innate and adaptive mechanisms of immune response, axonal transport, traumatic brain injury, and recessive general-body neurodegeneration [6, 8].

In the skin, the* UCHL1*/PGP 9.5 is strictly connected with nerve terminals; however, several studies also demonstrated its expression by neuro-immune-endocrine cells of the skin [13]. Basal and suprabasal keratinocytes, melanocytes, and Merkel and Langerhans cells may contain* UCHL1*/PGP 9.5 [13–15].* UCHL1*/PGP 9.5 comes from two independent expression sources, primarily from CNS, but secondarily from cutaneous-derived sources, and both meet in the skin [14–17]. Thus,* UCHL1*/PGP 9.5 seems to well fitful criteria of a marker, which could identify the nonneuronal “synaptic-like” connections proposed by Boulais and Misery [18]. Several studies demonstrated modification of PGP 9.5 axonal nerve terminals and neuro-immune-endocrine cells in the skin during chronic cutaneous neuroinflammation [19]. Those abnormalities provide induction of molecular changes, which in advanced skin lesions might resemble immune-mediated tissue injury and engage psoriatic keratinocytes in transmission of itch [20–24].

We aimed to investigate the connection between skin innervations in psoriatic individuals and itch sensation. We also have tried to elaborate the potential role of* UCHL1* gene as a new neurobiological marker of itch.

## 2. Material

### 2.1. Patients

Twenty psoriatic patients (20% females and 80% males) were included into the study. Their age ranged from 24 to 65 years (mean 44.1±12.5 years). None of the included patients had received any local or systemic therapies for at least three months prior to entering the study. Individuals with other cutaneous or systemic diseases which might interfere with study results were excluded. Patients were divided into two groups in relation to their itch status: with (Itch+) and without (Itch-) itch (n=14 and n=6, respectively). The control group consisted of 20 healthy individuals (60% females and 40% males) who underwent a routinely scheduled correction during plastic surgery. The age of control group ranged from 30 to 79 years (mean 56.3±13.0 years). All patients provided written informed consent, while study protocol was approved by the Ethics Committee at the Wroclaw Medical University (decision 669/2011) and was carried out in accordance with the principles of the Helsinki Declaration.

### 2.2. Reference Material

The total brain RNA (Agilent Technologies, USA) from human postmortem and human brain hippocampus (Clontech Laboratories, USA) were used for* UCHL1* gene transcript and PGP 9.5 protein detection.

## 3. Methods

### 3.1. Clinical Parameters

Psoriasis Area Severity Index (PASI) was used as the parameter to assess the severity of psoriasis. The mean PASI in analyzed patients was 15.7±10.2 points (range: 6.4–44.0 points). The Visual Analogue Scale (VAS) score was used for evaluation of maximal itch intensity within 24 hours prior to skin biopsy (mean: 3.9±2.8 points; range: 0–8 points).

### 3.2. Skin Biopsies: Preservation and Fixation

The 5 mm punch biopsies were taken from nonlesional and lesional skin of patients with psoriasis. Using sterile scissors biopsies were divided and primary one was immediately preserved in RNA-later (Qiagen, USA) solution and transferred to the -80°C. The second part of a biopsy was fixed in 4% paraformaldehyde (PFA) solution in 0.1 M phosphatase buffer saline (PBS) for 12 hours and transferred to the 30% sucrose (POCH, Poland) solution in 133 mM of Sörensen buffer for another 24 hours at 4°C. After fixation, biopsies were immediately immersed in optimum cutting temperature medium (O.C.T., CellPath, United Kingdom) and 15 *μ*m frozen skin sections were prepared (Superfrost Ultra Plus, Thermo Scientific™, Germany) using cryostat (Leica, Germany) and stored at -20°C for further analysis.

### 3.3. Cell Cultures

Human immortalized keratinocyte cell line HaCaT (ACTT, USA) were cultured in DMEM (Biowest, USA) growth medium supplemented with 10% FBS (Gibco, USA) and 1% PSA (Sigma-Aldrich, USA) solution at 37°C in a humidified atmosphere of 5% CO_2_ and 95% air. Cells after 70–80% confluence were incubated with 0.05% trypsin/EDTA (IITD PAN, Wroclaw, Poland) for 5 min., passaged, seeded at 2x10^5^density in 6-well plate, and preserved for further experiments. For gene expression analyses, cells were suspended in 1 ml of Trizol (Invitrogen, USA) and immediately transferred to -80°C. For protein analysis, cells were lysed using 1 ml of RIPA buffer and transferred to -20°C. Finally, for immunofluorescence imaging, cells were seeded at 96-well plate and fixed in 4% PFA for 10 min. at RT and 100*μ*l of PBS per well was added before immunofluorescence protocol.

### 3.4. RNA isolation and Reverse Transcription

The total RNA was isolated from skin biopsies embedded in RNAlater (Ambion, USA). The skin fragments were homogenized in Trizol (Invitrogen, USA) and introduced to the Tissue Fibrous Kit (Qiagen, USA) protocol. The DN-ase I treatment was applied to exclude possible contaminations and prevent hybridization of primers and probes to gDNA. Additionally, commercially available One-Step PCR Removal Inhibitor Kit (Zymo Research, USA) was also used for purification of RNA from possible PCR skin inhibitors. The quality and quantity of RNA after extraction were estimated using Nanodrop (Eppendorf, Germany) and only samples with 260/280 nm absorbance coefficient in the range between 1.8 and 2.1 were further used. The RNA samples were incubated for 10 minutes at 65°C and immediately transferred on ice to minimize formation of RNA secondary and tertiary structures before reverse transcription. The 500 ng of total RNA was reverse transcribed on cDNA using First-Strand cDNA Synthesis Kit for RT-PCR (Roche, Germany).

### 3.5. Probe and Primer Design

Primers and probes for human* UCHL1* gene and housekeeping* ACTB*, as well as universal probe library (UPL) hydrolysis probes, were designed using ProbeFinder 2.48 (Roche, Germany) software, UCHL-1 forward: CCTGAAGACAGAGCAAAATGC and reverse: TGTCATCTACCCGACATTGG (Genomed, Poland) with detection system of UPL 27 probe amplified 95 nucleotide region at the border of constitutively expressed exons (4/5). Reference sequence for* UCHL1* gene was NM_004181.4 (NCBI, USA).

### 3.6. Relative Gene Expression by Real-Time PCR (RT-PCR)

The RT-PCR reaction was performed in Roche LightCycler 480 II Thermocycler (Roche, Germany) in a volume of 10 *μ*l. The reaction volume consisted of 5 *μ*l of LightCycler UPL ProbeMaster (Roche, Germany), 0,5 *μ*M forward and reverse primers, and 0.2 *μ*M UPL probe 27 and 1 *μ*l cDNA. The reaction conditions were as follows: preincubation at 95°C by 10 min., 50 cycles of amplification step: 95°C by 15 s for denaturation, 58°C by 1 min. for elongation step, and 10 s at 72°C for detection and next the plate was cooled at 40°C for 10 s. The relative gene expression was presented using 2^-ΔΔCt^ method.

### 3.7. Immunofluorescence

The frozen skin sections and HaCaT fixed cells underwent the same immunofluorescence protocol with additional preincubation step for skin frozen sections with incubation for 10 minutes in solution of 5% acetic acid (POCH, Poland) in PBS and blocking step for cells and sections in blocking solution (BS): 3% bovine serum albumin (BSA) (LabEmpire, Poland), 5% normal donkey serum (Jackson Immunoresearch, USA), 0.05% Tween 20 (Sigma-Aldrich, Germany), and 0.01% Triton-X100 (Sigma-Aldrich, Germany) in PBS with incubation time of 1 hour at 4°C. After blocking step and 3x PBS rinsing, cells and sections were incubated with primary mouse anti-human PGP 9.5 antibody (13C4 clone, Ultraclone, United Kingdom) diluted in PBS (1:500) for 24 hours at 4°C temperature. Negative control was prepared by omitting the primary antibody and IgG-Alexa Fluor 488 isotype control (BD Bioscience, USA). Next day, sections were rinsed three times in PBS for 5 min. and incubated for 2 hours at RT in dark conditions with the secondary donkey anti-mouse Alexa Fluor 546 H+L (A-10036, Invitrogen, USA) antibody in PBS (dilution 1:500). In turn, donkey anti-mouse Alexa Fluor 488 H+L (A-21202, Invitrogen, USA) was used for detection of PGP 9.5 in HaCaT cultures with the same conditions. The immunofluorescence mounting medium with DAPI (Invitrogen, USA) was used for nucleus counterstaining.

### 3.8. Western-Blot

Skin biopsies were homogenized in 400 *μ*l of cold PBS using electric tissues homogenizer (ProScientific, USA). After disruption, 400 *μ*l of 2X Radioimmunoprecipitation Assay Buffer (RIPA, 50 mMTris-HCl, 150 mMNaCl, 0.1% SDS, 1% NP-40, 0.5% DOC, and pH=8.0) was added with proteinase cocktail inhibitor (Sigma-Aldrich, Germany). Next, probes underwent centrifugation (15 min. 10x10^3^g, 4°C) and supernatants were collected. The protein concentration was determined using a BCA Protein Assay Kit (Pierce, IL, USA,). The 40 *μ*g of protein lysates was mixed with Laemmli's loading buffer (4:1) and denatured by incubation at 95°C for 5 min. and immediately transferred on ice. As reference material, the 3 *μ*g of normal human hippocampus (Clontech Laboratories, USA) was used. The 12% SDS-PAGE gel electrophoresis was performed and protein transfer was verified by Coomassie Brilliant Blue (Sigma, USA) staining. Next, semidry electrotransfer from gels to PVDF and Whatman membranes (0.45 *μ*m, Millipore, USA) was performed for one hour (Bio-Rad, USA). In the next step, blotted membranes were hydrated by rinsing 3 times for 5 min. in solution of Tris-buffered saline with 0.05% Tween 20 (TBST) and incubated with 1% casein solution in TBST (0.1% Tween-20) at RT for 1 hour. Next, the primary mouse anti-human PGP 9.5 (1:10000, Utraclone, 13C4 clone), the same antibody used for immunofluorescence, was diluted in TBST with 1% casein and incubated overnight at 4°C. Rabbit polyclonal anti-human *β*-actin (1*μ*g/ml, 1:2000, Cat. num.: SC-7210, Santa Cruz Biotechnology, USA) was used as reference. Next day, membranes were incubated with secondary antibodies conjugated with horseradish peroxidase-HRP diluted in blocking solution. Polyclonal goat anti-mouse-HRP (1:1000, Cat. num. P0447, Dako, Denmark) was used for PGP 9.5, while polyclonal goat anti-rabbit-HRP (1:1000, Cat. num.: P0448, Dako, Denmark) was used for *β*-actin detection. The chemiluminescence detection kit (SuperSignal™ West Pico Chemiluminescent, Cat. num.: PI-34078, Thermo Scientific, USA) was used for bands visualization.

### 3.9. Image Analysis

For frozen skin sections, immunofluorescence detection was carried out using the fluorescence microscope (Zeiss, Germany) with constant exposure conditions (1000 ms), microscope camera settings (Zeiss, Germany) at Zeiss Vision Image (ZVI) format using AxioVision software (Zeiss, Germany). In turn, positive immunofluorescence in HaCaT cells was detected and analyzed using the scanning confocal microscopy (Zeiss, USA). All fluorescence ZVI images were converted into the 8-bit RGB TIFF image files and transferred to the Fiji software (Fiji, ImageJ, National Institute of Health, Bethesda, USA).

### 3.10. Statistical Analysis

All statistical analyses were performed using Statistica 12.0 software (Dell Software, USA). Differences between samples from compared patient groups were verified using Mann–Whitney* U* test. Correlation data were analyzed by Spearman rank correlations test. Values of* p *<0.05 were considered statistically significant.

### 3.11. Data Presentation

The presented graphs were performed using GraphPad Prism 5.0 (GraphPad Software Inc., La Jolla, CA, USA), while figures were prepared by LibreOffice 5.0 Software (The Document Foundation, Germany).

## 4. Results

**Figure 1:**
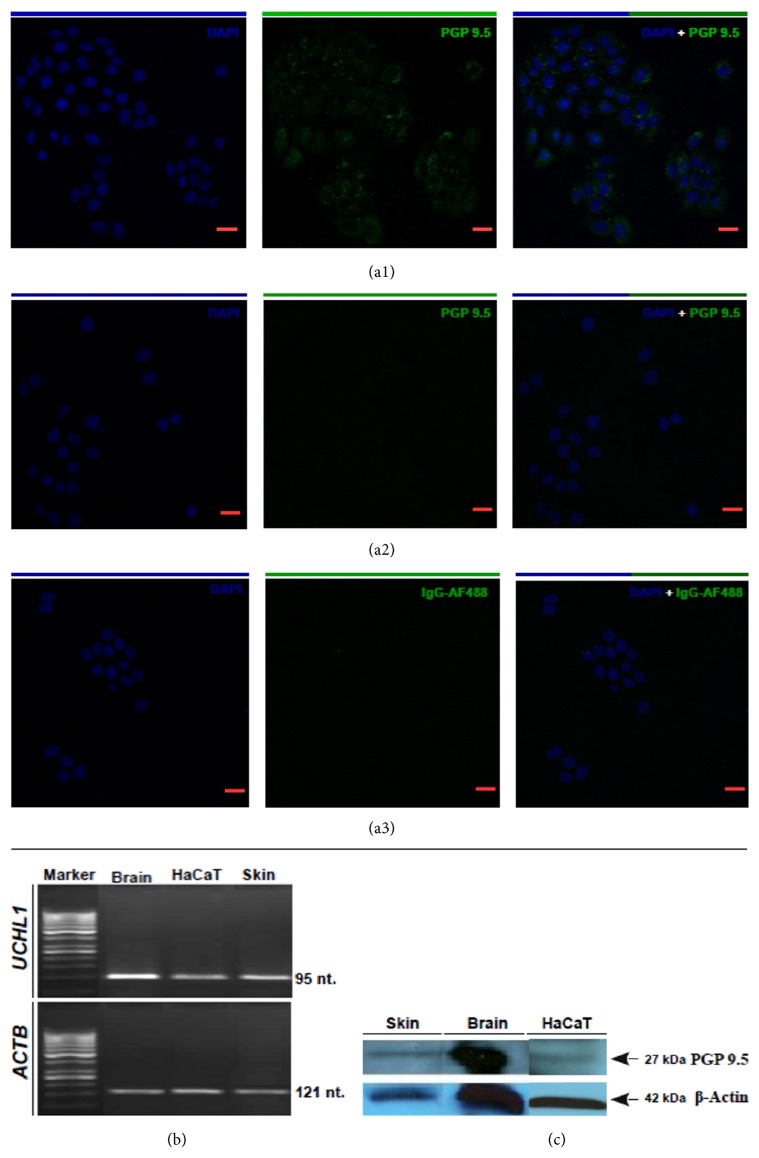
**Expression of* UCHL1* gene and PGP 9.5 protein in skin punch biopsies, HaCaT keratinocyte cell line, and brain reference material.** Confocal scanning microcopy using HaCaT keratinocyte cell line presents images of cytoplasmatic expression of PGP 9.5 protein (a1). Furthermore, omission of primary antibody against human PGP 9.5 (a2) as well as IgG-AF488 isotype control does not result in any specific signal (a3). Scale bars represent 20*μ*m.* UCHL1* expression at mRNA level was observed in HaCaT cell line, normal skin, and was confirmed in brain reference material. Forward and reverse primers amplificate 95nt sequence at the border of exons 3 and 4 of* UCHL1* gene detected using FAM-labeled UPL 27 probe.* ACTB* was used as housekeeping gene using VIC-labeled probe and amplificate 121nt fragment. All amplified material was visualized using gel electrophoresis (b). Protein expression was verified using Western-blot with the same mouse anti-human PGP 9.5 antibody and gave specific 27 kDa band in the skin, brain, and HaCaT cell line, respectively (c).

Results of our investigations demonstrate cutaneous expression of* UCHL1*/PGP 9.5:* UCHL1* gene transcript, as well as PGP 9.5 protein expression in total skin biopsy samples. We detect PGP 9.5 by basal (BKs) and suprabasal (SBKs) epidermal keratinocytes as well as DRG axonal nerve terminals and dermal nerve fibers, respectively (graphs and images presented below). Furthermore, barely but stable expression within immortalized HaCaT cells was also demonstrated (Figure 1: (a1)–(a3), (b), and (c)). All reagents used for detection of* UCHL1* transcript and PGP 9.5 protein were confirmed in the human brain reference material (Figure 1(c)).

### 4.1. PGP 9.5 Distribution and Expression in the Skin of Controls and Psoriasis Patients

**Figure 2:**
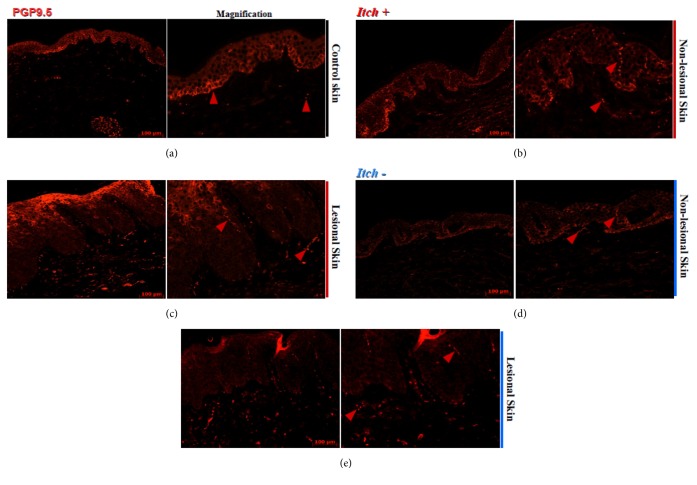
**PGP 9.5 distribution and expression in axonal nerve terminals and dermal nerve fibers in skin samples of healthy individuals and nonlesional and lesional skin of psoriasis with or without itch.** PGP 9.5 of epidermal axonal nerve terminals and dermal fibers in normal healthy skin analyzed by indirect immunofluorescence staining on frozen skin sections (a). The nonlesional (b) and lesional skin (c) with itch (*Itch+*), in relation to nonlesional (d) and lesional skin (e) without itch (*Itch*-) from psoriasis patients. Axonal nerve terminal and dermal nerve fibers marked with red arrows. Scale bars represent 100 *μ*m.

The PGP 9.5 somatosensory system was detectable in the skin of both healthy and psoriatic subjects. In normal epidermis, PGP 9.5 signal was present in axonal nerve terminals and dermal nerve fibers. The single nerve terminals penetrating all epidermal layers were detected only occasionally in stratum spinosum or granulosum. We observed single nerve terminals in normal dermis, which in some subjects crossed dermal-epidermal border. A perivascular PGP 9.5 expression was also observed in dermal microvascular network, sweet glands and dermal papillae (Figure 2(a)). Furthermore, cytoplasmic expression of PGP 9.5 was demonstrated in normal BKs and considered as single or group arranged cells. Normal SBKs demonstrate almost complete absence of PGP 9.5 with some barely and heterogeneous expression via single epidermal keratinocytes (Figure 2(a)). The nonlesional epidermis showed numerous nerve terminals, while some of them even reached stratum corneum, with strong expression of PGP 9.5 within BKs (Figures 2(b) and 2(d)). In turn, PGP 9.5 expression in psoriatic lesions was more diversified and demonstrated gradual expression patterns. The PGP 9.5 expression was observed in BKs with free PGP 9.5 epidermal nerve terminals or without detectable expression within BKs but with tendency to turn over from weak PGP 9.5 nerve ending expression to strong PGP 9.5 expression by SBKs. Nerve terminals in skin lesions seemed to be more elongated in comparison to nonlesional skin and healthy individuals (Figures 2(c) and 2(e)).

### 4.2. *UCHL1* Gene Expression in the Skin Biopsies

**Figure 3:**
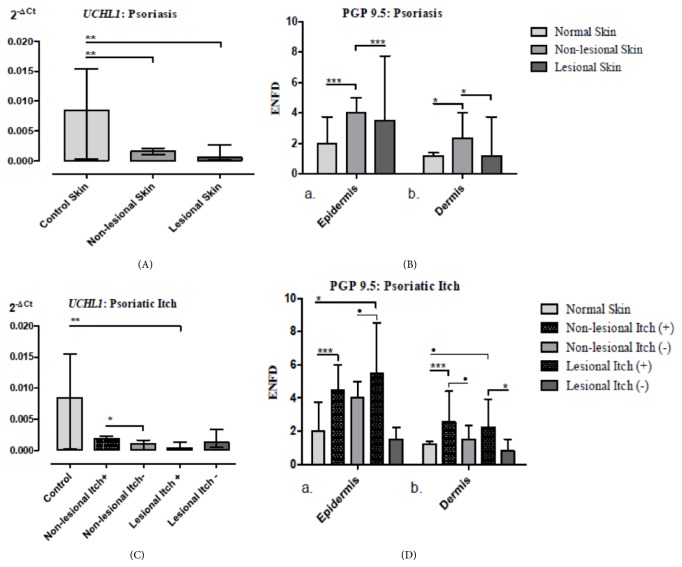
**Graphs summarizing results of relative* UCHL1* gene expression and PGP 9.5 expression within epidermal axonal nerve terminals and dermal fibers in the skin of healthy control and psoriatic patients with division into with (*Itch*+) and without (*Itch*-) itch.** The relative expression of* UCHL1* gene in psoriasis (A) and epidermal nerve fiber density (ENFD) of PGP 9.5 axonal nerve terminals and dermal nerve fibers in nonlesional and lesional skin of psoriasis (B) along with the division into with (itch+) and without (itch-) itch ((C), (D)).

The expression of* UCHL1* gene in skin biopsies of psoriasis patients was downregulated in comparison to group of healthy individuals (Figure 3(A)).* UCHL1* expression in nonlesional psoriatic skin (mean: 0.002; SD: ±0.0008, p<0.001) was significantly lower in comparison to control group (mean: 0.008; SD: ±0.007). The psoriasis patients without itch in nonlesional (mean: 0.001, SD: ±0.00065) and lesional skin (mean: 0.002, SD: ±0.001) did not demonstrate significant differences of* UCHL1* expression compared with control group (mean: 0.008, SD: ±0.007). However, nonlesional skin without itch (mean: 0.00098, SD: ±0.0007) demonstrated significantly lower* UCHL1 *expression in comparison to nonlesional skin with itch (mean: 0.0019, SD: ±0.0007, p=0.04). While skin lesions with itch (mean: 0.0018, SD: ±0.0035) demonstrate significantly decreased* UCHL1* expression compared to control group (mean: 0.008, SD: ±0.007, p=0.02), such differences were not observed between psoriatic lesions without itch (mean: 0.00174, SD: ±0.0016) and normal skin (mean: 0.008; SD: ±0.007, p=0.15) (Figure 3(C)).

### 4.3. Density of PGP 9.5 Epidermal Nerve Terminals and Dermal Nerve Fibers

The numbers of epidermal PGP 9.5 axonal nerve terminals (mean: 4.5, SD: ±1.47; p<0.001) and dermal nerve fibers (mean: 2.53, SD: ±1.44; p<0.01) were significantly increased in nonlesional skin of patients with psoriasis compared to epidermis (mean: 2.6, SD: ±1.39; p<0.001) and dermis (mean: 1.21, SD: ±0.53; p<0.01) of healthy individuals (Figure 3(B)). In relation to psoriatic skin lesions, the number of epidermal PGP 9.5-positive axonal nerve terminals (mean: 4.5 SD: ±4.5; p=0.07) and dermal fibers (mean: 2.1, SD: ±1.8; p=0.04) seems to be higher than their epidermal (mean: 2.6, S.D.: ±1.4) and dermal (mean: 1.2, SD: ±0.5) control counterparts (Figure 3(A)). In relation to itch, we observed significantly higher number of epidermal nerve terminals (mean: 4.93, SD: ±1.21, p<0.001) and dermal nerve fibers (mean: 2.94, SD: ±1.21) in nonlesional skin with itch compared to healthy subjects (mean: 1.21, SD: ±0.53; p=0.001) (Figures 2(a) and 2(b); Figure 3(D)). We also observed significant differences between nonlesional epidermis with itch (mean: 4.93, SD: ±1.21) compared to nonlesional skin without itch (mean: 3.50, SD: ±1.64; p<0.05). In turn, itchy nonlesional dermis (mean: 2.93, SD: ±1.42) seems to have more fibers compared to that without itch (mean: 1.58, SD: ±1.04), but the difference did not reach the statistical significance (p=0.07) (Figures 2(b) and 2(d)). Moreover, epidermis (mean: 5.86, SD: ±4.74) of itchy lesions, similarly to dermis (mean: 2.56, SD: ±1.83), had higher distribution of axonal nerve terminals and fibers compared to epidermis (mean: 1.50, SD: ±1.05; p=0.04) and dermis without itch (mean: 1.03, SD: ±1.02; p=0.07); however, statistical significance was achieved only regarding the epidermis (Figures 2(c) and 2(e); Figure 3(B)). Furthermore, we observed the positive correlation between VAS score and both PGP 9.5 epidermal axonal nerve terminals (r=0.47; p<0.05) and number of dermal nerve fibers (r=0.42; p<0.05) in lesional psoriatic skin (data not shown). In addition, reverse correlation between disease severity (PASI score) and both PGP 9.5 expression in lesional epidermis (r=-0.51; p<0.05) and dermis (r=-0.45; p<0.05) was also observed.

### 4.4. PGP 9.5 Basal (BKs) and Suprabasal (SBKs) Keratinocyte Expression

**Figure 4:**
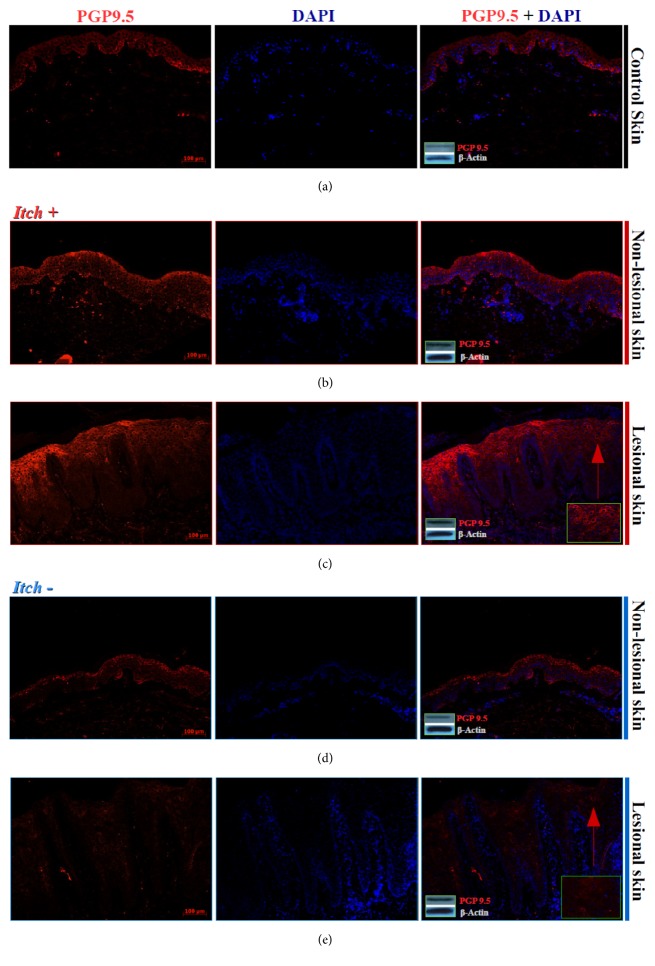
**Expression of PGP 9.5 by epidermal keratinocytes in control skin and nonlesional and lesional skin with (Itch+) and without (Itch-) itch.** Immunofluorescence images of epidermal expression of PGP 9.5 in control healthy skin was found mostly in basal keratinocytes (BKs) with weak detection by suprabasal keratinocytes (SBKs) (a). Further images represent BKs and SBKs PGP 9.5 expression in nonlesional and lesional samples with ((b), (c);* Itch+*) and without ((d), (e);* Itch*-) itch. Inserts: western-blot analysis of PGP 9.5 expression of the representative skin samples of two controls as well as two patients with (*Itch+*) and two without (*Itch*-) itch, respectively. Representative bands of PGP 9.5 (27kDa) and *β*-actin (42kDa) endogenous control are listed in the lower right corners of merged images.

**Figure 5:**
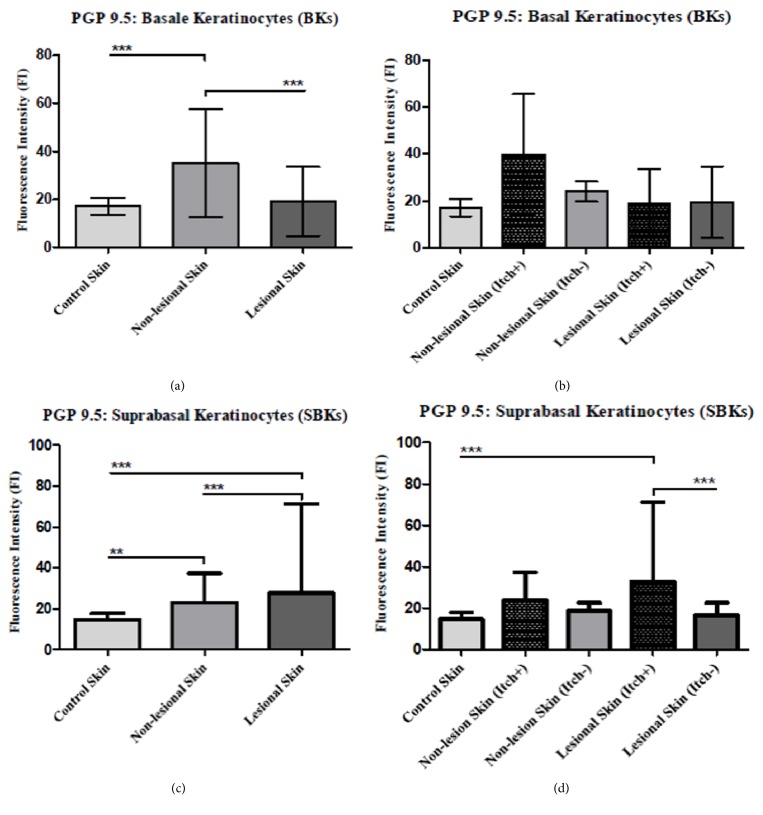
**Graphs summarizing results of semiquantitative fluorescence intensity (FI) of PGP 9.5 expression by BKs and SBKs in the skin of healthy controls and psoriatic patients with* (Itch-)* and without* (Itch-)* itch.** The semiquantitative fluorescence intensity of BKs and SBKs in healthy control persons and nonlesional and lesional skin of psoriasis patients ((a), (c)) with (itch+) and without (itch-) itch ((b), (d)).

In general, we demonstrated significantly higher cytoplasmic expression of PGP 9.5 protein by basal (BKs) as well as suprabasal keratinocytes (SBKs) than controls (Figure 4). The BKs of nonlesional skin demonstrated significantly higher expression of PGP 9.5 protein (mean: 35.1, SD: ±22.6; p=0.001), and SBKs did (mean: 21.6, SD: ±7.0: p=0.0001) in comparison to BKs (mean: 17.2, SD: ±3.6) and SBKs (mean: 14.3, SD: ±2.2) of control group (Figures 4(a), 4(b), and 4(d); Figures 5(a) and 5(b)). Furthermore, nonlesional PGP 9.5-expressing SBKs positively correlated with VAS (SBKs: r=0.49,p=0.02), while, regarding PGP 9.5-expressing BKs, no significant correlation with VAS was noted (BKs: r=0.35, p=0.13). In lesional skin, significant discrepancies of PGP 9.5 expression concerned only SBKs (mean: 32.5, SD: ±17.2, p<0.001) when compared to controls (mean: 14.3, SD: ±2.2) (Figure 5(b)), while psoriatic BKs (mean: 19.2, SD: ±14.3; p=0.54) and controls (mean: 14.3, SD: ±2.2) demonstrated comparable PGP 9.5 expression (Figures 4(a), 4(c), and 4(e); Figure 5(a)). PGP 9.5 expressing SBKs in psoriatic lesions positively correlated with itch regarding VAS score (SBKs: r=0.64, p=0.002). The differences were also observed between nonlesional BKs (mean: 26.1, SD: ±18.4; p<0.001) compared to BKs of psoriatic lesions (mean: 18.2, SD. ±10.38) (Figures 4(b), 4(c), 4(d), and 4(e); Figure 5(a)). SBKs of cutaneous lesions (mean: 23.37, SD: ±15.23; p=0.007) expressed PGP 9.5 at significantly higher level compared to nonlesional skin (mean: 17.93, SD: ±6.34) counterparts (Figure 5(b); FI: Figures 4(c), 4(e), 4(b), and 4(d)). Furthermore, only SBKs of itchy skin lesions (mean: 38.91, SD: ±16.72; p=0.006) presented significantly increased expression of PGP 9.5 in comparison to lesional skin without itch (mean: 17.42, SD: ± 3.88) (Figures 4(c) and 4(e); Figure 5(d)).

## 5. Discussion

Expression of* UCHL1*/PGP 9.5 system is observed in cells with neuro-immune-endocrine activity and DRG neurons, in which terminals indirectly interplay with epidermal keratinocytes and are fundamental organizers of the cutaneous somatosensory complex [25, 26]. Experimental sciatic nerve injury in animals demonstrated rapid downregulation of* UCHL1*/PGP 9.5 expression by nerve terminals, whereas its amounts became compensated mainly by Langerhans cells (LCs) and Merkel cells (MCs), respectively [15, 25, 27]. However, several studies indicated that expression of PGP 9.5 may also be considered normal epidermal keratinocytes and dermal fibroblasts [28]. In turn, rapid induction of* UCHL1*/PGP 9.5 by cells of nonneuronal origin, like keratinocytes, have been discussed and multiple scenarios were proposed [28, 29]. The keratinocytes engagement in itch-mediated signals was proved in a number of studies using* in vitro* assays [26, 30]. In turn, studies from clinics demonstrated involvement of nervous system in psoriasis, and animal models confirmed these relations [25, 31–33]. In context of current studies, reverse correlation between higher PGP 9.5 protein and its lower* UCHL1* gene expression was observed, with emphasis on the lowest* UCHL1* in itchy lesions. Thus, essential question has arisen of whether decreased* UCHL1* expression in psoriasis might be linked with impaired function of neuro-immune-cutaneous milieu. LCs impaired density is usually observed* in vivo* in pain-related syndromes. Keratinocytes and PGP 9.5^+^ LCs closely cooperate via direct contact with itch-mediating PGP 9.5^+^, CGRP^+^ neurons [34]. CGRP is a well-known cell-immunity modifier, which abolishes LCs antigen presentation. Its increased amounts were noted to be released from nerve terminals in psoriasis [34, 35]. Opposite results were obtained by Sebastian et al., who demonstrated that, in electrically induced wound injury model, expression of* UCHL1* gene and PGP 9.5 was significantly increased at nerve terminals, but also in MCs together with other commonly applied neuronal markers [36]. Keratinocytes in psoriatic lesions are neuroinflammatory cells and induced expression of other neurorelated proteins may reflect their injured conditions [33]. Furthermore, PGP 9.5 positive branches of DRG neurons are significantly increasing in the presence of infiltrating cells like eosinophils, both* in vitro* and* in vivo* in atopic dermatitis, and this mechanism should not be negated in psoriasis [35]. In psoriasis, number of proinflammatory mediators, such as prostaglandins, is elevated, whereas their reactive lipid compounds contribute to molecular modification of covalently bounded cysteine residues in structure of PGP 9.5, e.g., in brain studies [37]. Whether inducible expression of* UCHL1*/PGP 9.5 system in nonneuronal cells like keratinocytes may be a sign of appearing molecular dysfunction and participate in transmission of subjective sensations still needs to be confirmed. However, Johansson et al. indicated that single keratinocytes in suprabasal compartments of normal epidermis exhibit PGP 9.5 expression [28, 29]. Inducible expression of* UCHL1* was seen* in vitro* in primary outgrowing keratinocytes arising from psoriasis patients, while in microdissected skin it was only slightly elevated [38]. In turn, Eiding et al. studies presented that* UCHL1* gene was significantly increased in nonlesional and lesional keratinocytes derived from epidermis of psoriasis patients, especially in those subjected exposed to UVB irradiation [32]. The series of further studies demonstrated that* UCHL1*/PGP 9.5 system may regulate melanogenesis dependent on agouti signal protein (ASIP, ASP in mice). ASIP is a naturally widely expressed tissue inhibitor for melanocortin-1 receptor (MC1R) and is located in so-called “ITCHy” locus. The locus contains principal proteins and transcription factors responsible for skin development. Moreover its name arises from being situated there HECT-ITCH ligase E3 enzyme also belonging to ubiquitin system. Its genetic knockdown results in development of non-agouti-lethal 18H mice with spectrum of immune dysfunction and main behavior of uncontrolled scratching of the skin [39, 40] Melanocyte cell line treated by ASP recombinant demonstrates rapid upregulation of* UCHL1,* together with genes responsible for axonal guidance and neuronal development [41]. Moreover, Young Seo and coworkers showed that overexpression of* UCHL1*/PGP 9.5 by melanocytes resulted in microphthalmia-associated transcription factor (MTIF) ubiquitination and degradation, with dramatic reduction of pigmentation [42]. MITF consists of critical protein responsible for melanocytes development and melanogenesis, so it is possible that phototherapy-induced release of* UCHL1*/PGP 9.5 via melanocytes may enhance its horizontal transfer to the neighboring keratinocytes using exosome-dependent pathway [43]. Melanocytes in the epidermis are located in the basal layer, while their prominent and long dendrites penetrate to the upper epidermal compartments participating in melanin transfer to the surrounding keratinocytes [44]. In psoriasis, melanocytes are elevated; however, MITF expression was noted to be comparable to controls [45]. The attention should be also focused to other studies where model of experimental skin injury demonstrated participation of PGP 9.5 with other axonal transport factors in synaptic cargo vesicles from DRG bodies to the periphery [46]. Thus, whether stable expression of PGP 9.5 protein within human epidermal keratinocytes is MITF-independent and whether intracellular communications are engaged should be further elucidated.

The epigenetic cytosine-phosphate-guanine (CpG) DNA hypermethylation of* UCHL1*/PGP 9.5 promoter is a well-known mechanism responsible for partial or complete silencing of the expression within tissues and was well demonstrated in melanoma cell lines and patients, respectively [47]. Several studies using different spectrum of molecular techniques underlined infections, oxidative stress, or immune-mediated disorders as possible factors down- or upregulating* UCHL1*/PGP 9.5 in PNS cells as well as in nonneuronal cells [10, 48, 49]. Using siRNA technique,* UCHL1*/PGP 9.5-expressing cell lines showed more than 200 downregulated and comparable number of upregulated genes engaged in most cellular processes, also those responsible for molecular background of psoriasis [50]. So, molecular mechanisms of protein ubiquitination seem to be deregulated in inflammatory-associated diseases and other ubiquitin-related enzymes participating in signaling pathways are analyzed to better explain their roles in psoriasis disease [51]. Furthermore, new findings connect neurodegenerative symptoms and neuropathic pain with activation of ubiquitination pathways; however, sensations, such as itch, are still poorly explained in that context [11]. Increased numbers of PGP 9.5 axonal nerve terminals and dermal nerve fibers in the skin are considered as the main itch transmitting factor and our data confirmed association of altered PGP 9.5 neuroepidermal profile with itch. Nakamura et al. have demonstrated that psoriasis patients have higher number of PGP 9.5 nerve terminals in the epidermis which positively correlated with itch intensity [52]. Similar results were observed by Takamori et al.; however, in both studies analyses were only restricted to the diseased skin [53]. Johansson and colleagues indicated that PGP 9.5 nerve terminals are altered only in the skin with lesions, while nonlesional skin resembled the samples from controls. PGP 9.5 in psoriatic lesions were more frequently presented on the dermal-epidermal border and showed restricted pattern in epidermis, which is consistent with our observations [54]. In our study, the number of PGP 9.5 terminals in psoriatic lesions was comparable with healthy controls; however, those in epidermis and dermis seemed to be more elongated. In nonlesional skin, PGP 9.5 terminals were predominantly present in the epidermis. However, patients with itch had significantly higher number of PGP 9.5 nerve terminals in the epidermis and fibers in the dermis in comparison to the skin without itch and healthy control group. We also observed that number of PGP 9.5 axonal nerve terminals in the epidermis and dermis of lesional skin positively correlated with VAS score. Pergolizzi et al. demonstrated that epidermal PGP 9.5 nerve terminals in long-established skin lesions of psoriasis may undergo almost complete reduction. They postulate gradually occurring changes in the psoriatic lesions at initial phase of psoriasis with increased innervations and nerve projections through all epidermal layers, to almost complete reduction at advanced stages [55]. These results partially explained our observations and may depend on exacerbation phase of psoriasis [56]. The number of PGP 9.5 nerve terminals was also recorded as increased in samples taken from scalp of psoriatic patients; however, no relation with itch was found [57]. The participation of C-fibers in psoriatic itch was also confirmed by transcutaneous electrical nerve stimulation, where mean of somatosensory threshold level was significantly higher in itchy lesional skin [58]. Significantly altered PGP 9.5 nerve terminals were observed in group of stress exposed healthy volunteers. Acute psychoemotional stress conditions may increase the number of cutaneous PGP 9.5 nerve terminals. Thus, decreased neuropsychological conditions and skin neuroinflammation may also exacerbate PNS in psoriasis [59, 60].

## 6. Conclusions

PGP 9.5 profiling of axonal nerve terminals in skin samples of patients with psoriasis may constitute a useful method for assessment of itch. In turn, our studies for the first time indicated that increased itch-signal transmission might be a consequence of nonneuronal synapse dysfunctions. The interaction between nerve terminals penetrating epidermis and their terminations within neuro-immune-endocrine cells in psoriasis might consist one of the possible mechanisms. Thus, our findings suggest that disordered interplay between axonal transport and epidermal keratinocytes, as well as impaired ubiquitylation mechanisms, may constitute a new molecular player in itch transmitting pathways.

## Data Availability

All data used to support the results of this study are included within the article.
